# Implanted *β*-Tricalcium Phosphate Blocks Can Function as a Placeholder in Recurrent Giant Cell Tumor of Bone

**DOI:** 10.1155/2020/2571940

**Published:** 2020-01-16

**Authors:** Akio Sakamoto, Takeshi Okamoto, Shuichi Matsuda

**Affiliations:** Department of Orthopaedic Surgery, Graduate School of Medicine, Kyoto University, Japan

## Abstract

The giant cell tumor of bone (GCTB) is a locally aggressive tumor. Reconstruction methods using *β*-tricalcium phosphate (*β*-TCP) blocks with strong compression resistance in and around the knee joint for GCTB have been reported. Among six cases of GCTB treated using this method, two recurrent cases revealed osteolysis, predominantly within the *β*-TCP block based on plain radiographs or computed tomography, while remodeled host bones were preserved. Implanted *β*-TCP blocks can function as a placeholder to preserve host bone in recurrent cases, leading to a higher probability of joint preservation.

## 1. Introduction

A giant cell tumor of bone (GCTB) is locally aggressive and typically occurs in patients aged 20 to 45 years. The distal femur and the proximal tibia are the most commonly affected sites, and the tumor occurs in the metaphyseal region and extends to the epiphysis [1].

GCTB is composed of neoplastic stromal cells and reactive multinucleated osteoclast-like giant cells [2]. A mutation in *H3F3A* is observed in neoplastic stromal cells [2]. Overexpression of receptor activator of nuclear factor-kappa B ligands by neoplastic stromal cells promotes the recruitment of reactive multinucleated osteoclast-like giant cells, leading to the osteolytic nature of GCTB.

The rate of GTCB recurrence after treatment with a simple curettage has been reported to range between 30% and 50% [1]. Several adjuvant methods have been reported in order to reduce the rate of recurrent GCTB after curettage, including the use of liquid nitrogen, phenol, ethanol, and polymethylmethacrylate bone cement. With these methods, recurrence rates have been reported to be in the range of 7% to 10% [1, 3, 4].

As previously reported, reconstruction with low-porosity *β*-TCP blocks can serve as a strut bone graft with its characteristic strong resistance in compression (hard-type *β*-TCP) (superpore hard-type: Pentax New Ceramics Division, Hoya Corporation, Tokyo, Japan) [5].

In the current article, recurrent cases were reported among six GCTBs treated by curettage and *β*-TCP block reconstruction. The recurring lesions were predominantly osteolytic in nature and located in the *β*-TCP block in both cases. The merits of the *β*-TCP block, in terms of the ease of detection of the lesion, and possible host bone preservation, are discussed herein.

## 2. Case Series

The 6 patients with GCTB in the vicinity of the knee consisted of 5 males and 1 female. The mean age at the first procedure was 37.0 years (range, 25 to 48 years). The location was the distal femur in two cases and the proximal tibia in four cases. The mean period of observation was 26 months (range, 12 to 42 months).

### 2.1. Case 1

**Figure 1:**
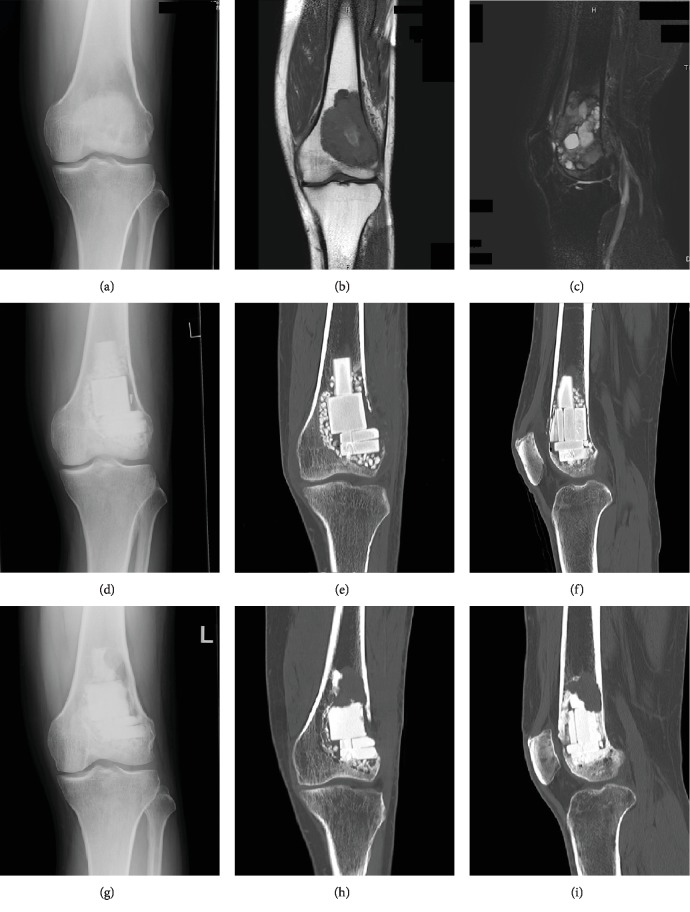
Giant cell tumor of bone in a 40-year-old male. Plain radiographs show ill-defined osteolysis in the distal femur (a). The lesion has homogenous low-signal intensity on T1-weighted images (b) and a heterogeneous intermediate- and high-signal intensity on T2-weighted images with fat suppression (c) on MRIs. The cavity after curettage is filled with *β*-TCP blocks and particles and is shown 1 month after the operation on plain radiographs (d) and CTs (e, f). Osteolysis is predominantly observed in *β*-TCP blocks on plain radiographs (g) and CT images (h, i) 1 year and 1 month after the operation.

A 40-year-old male reported enduring left knee pain for 6 months. Plain radiographs showed an ill-defined osteolytic lesion in the distal metaphysis and epiphysis of the femur. A diagnosis of GCTB was made based upon a needle biopsy. During the operation, after curettage of the lesion, a block-shaped, hard type of *β*-TCP was implanted in the cavity, and standard *β*-TCP particles were used to fill in additional spaces (Figure 1). One year and 1 month after the operation, osteolysis in the *β*-TCP appeared and was confirmed by computed tomography (CT). Cortical bone adjacent to the recurring lesion had thinned but was preserved. Curettage of the recurrent lesion was performed and *β*-TCP was implanted.

### 2.2. Case 2

**Figure 2:**
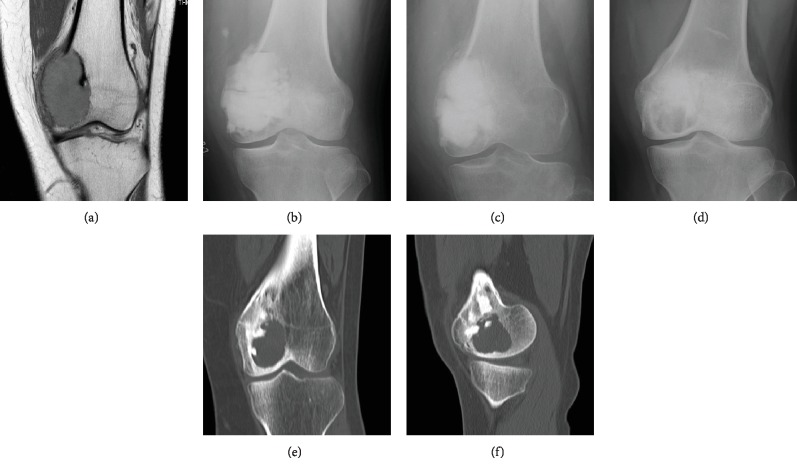
Giant cell tumor of bone in a 25-year-old female. The lesion has homogenous low-signal intensity on T1-weighted images (a). The implanted *β*-TCP is gradually remodeled and incorporated at 3 months (b) and 8 months (c) after the operation. Osteolysis is predominantly observed in *β*-TCP blocks in plain radiographs (d) and a CT image (e) 2 years and 5 months after the operation. The subchondral bone is preserved (f).

A 25-year-old female reported experiencing right knee pain for 2 months and had presented previously as a candidate for *β*-TCP block reconstruction [5]. Plain radiographs showed an osteolytic lesion in the distal femur, and magnetic resonance imaging (MRI) revealed that the lesion extended to the subchondral bone. After diagnosing GCTB by needle biopsy, curettage was performed, followed by the implantation of a *β*-TCP block in the cavity. Two years and 5 months after the operation, plain radiographs and CT revealed an osteolytic lesion adjacent to the joint. Osteosclerotic subchondral bone adjacent to the recurrent lesion was preserved (Figure 2). Curettage of the recurrent lesion was followed by another *β*-TCP implant.

## 3. Discussion

Reconstruction with *β*-TCP is used in our series of GCTB treatment. Bone cement is a common filler used after curettage of GCTB. However, treatment with bone cement may increase the risk of degenerative changes due to polymerization heat when the lesions are subchondral [6]. Degenerative arthritis after having filled a void with bone cement would require tumor endoprosthesis replacement, while an ordinal surface-type endoprosthesis for osteoarthritis would be difficult due to the existence of bone cement. During surgery to treat a recurrent lesion, implanted materials often need to be removed or sometimes partially removed to enable adequate access to the area. Bone cement is difficult to remove. These negative aspects of bone cement are not seen in *β*-TCP reconstruction.

In this series treated with *β*-TCP, recurrent GCTB lesions were osteolytic, predominantly within implanted *β*-TCP blocks as visualized using plain radiographs and shown even more clearly using CTs. In contrast, remodeled bone adjacent to the recurrent lesion was preserved. The implanted *β*-TCP appeared to function as a placeholder. In a previous report, *β*-TCP was shown to be resorbed by osteoclasts in the absence of normal osteoblast involvement in bone remodeling [7], which may explain the osteolytic findings in the *β*-TCP with recurrent GCTB. The possible deflection of osteolysis towards *β*-TCP was based upon imaging, but additional careful and thorough observation, such as *histomorphological* assessment and evaluation of osteoclast activity, would be necessary to verify this observation.

Deflection of osteolysis towards bone cement is not expected, as bone cement is not remodeled. Therefore, a recurrent lesion occurs in normal host bone surrounding the bone cement and increases the need for resection and tumor endoprosthesis replacement, compromising the likelihood of preserving joint structure. Deflection of osteolysis towards autograft/allograft bone would not be conclusive of recurrence. During bone remodeling of implanted autograft/allograft bone, radiographic appearance is sometimes difficult to distinguish from recurrence. In terms of detection of recurrence, *β*-TCP blocks would be more diagnostic of recurrence than would autograft/allograft bone.

Merits of bone cement include early postoperative rehabilitation and weight bearing [2]. The block-shaped *β*-TCP used in our series has high mechanical strength (hard type), with low porosities of between 65% and 71%, while standard-type *β*-TCP has porosities of 71% to less than 80% [8]. The compression strengths are more than 15 MPa (hard type) or more than 1.5 MPa (standard type). After hard-type *β*-TCP block reconstruction, rehabilitation is able to start after bone consolidation in the subchondral space, without having to wait for consolidation of the whole lesion; however, whole bone consolidation might be necessary for bone graft implantations.

In summary, after curettage of a GCTB, implanted *β*-TCP is the predominant site of osteolysis in recurrent cases, and detection is enabled using either plain radiographs or CT. Osteolysis, predominantly in *β*-TCP, increases the chance of preserving the joint because the *β*-TCP acts like a placeholder.
